# RNA and hematopoiesis

**DOI:** 10.1097/BS9.0000000000000128

**Published:** 2022-07-20

**Authors:** Jia Yu

**Affiliations:** a State Key Laboratory of Medical Molecular Biology, Haihe laboratory of Cell Ecosystem, Institute of Basic Medical Sciences, Chinese Academy of Medical Sciences, School of Basic Medicine Peking Union Medical College, Beijing, China; b Key Laboratory of RNA and Hematopoietic Regulation, Chinese Academy of Medical Sciences, Beijing, China; c Medical Epigenetic Research Center, Chinese Academy of Medical Sciences, Beijing, China

Hematopoietic stem cells (HSCs) can differentiate into all mature functional blood cells via hematopoiesis, but can also self-renew to replenish the progenitor cell pool. Both hematopoiesis and HSC renewal are strictly orchestrated processes to guard against dysregulation that could lead to life-threatening blood malignancies, such as leukemia. RNA regulatory networks have essential roles in ensuring normal hematopoiesis. Now, state-of-the-art, high-throughput sequencing technologies, including RNA-seq, CLIP-seq, ChIP-seq, ChIRP-seq, MeRIP-seq, are allowing us to gain comprehensive insights into the contribution of RNA regulatory networks to hematopoiesis and how they might be perturbed to promote malignancies.

In this issue, 5 leading scientists involved in RNA research, share with us their insights into the roles of RNA regulation in hematopoiesis and blood diseases. As detailed below, each has extensive experience in dissecting the cellular and molecular basis of RNA regulation and hematopoiesis, with their areas of interest covering RNA-binding proteins (RBPs), noncoding RNAs (ncRNAs), RNA chemical modifications, HSCs, and artificial intelligence (AI).

Prof Jia Yu is researching RNA regulation and hematopoiesis at the Institute of Basic Medicine Sciences, Beijing, China. Prof Yu’s research article entitled “KH-type splicing regulatory protein KHSRP regulates monocytic differentiation through combining transcriptional and posttranscriptional mechanisms” highlights the dual DNA- and RNA-binding activities of the chromatin-enriched (Che) RBP, KHSRP. Many RBPs are implicated in the transcriptional and posttranscriptional regulation of various biological processes. Among them, Che-RBPs accumulate in the chromatin fractions, where they likely regulate transcription. However, whether other RBPs also have chromatin-binding capacity was previously unknown. In an earlier work, Prof Yu performs a global screen to systemically investigate which Che-RBPs are expressed in hematopoietic cells and identify KH-type splicing regulatory protein (KHSRP) as a Che-RBP.^1^ Indeed, KHSRP involves in mRNA decay, alternative splicing, and miRNA biogenesis, as well as myeloid differentiation through facilitating miR-129 maturation.^2^ In this latest study, Prof Yu and colleagues analyze publicly available ChIP-seq, CLIP-seq, and RNA-seq datasets and pinpoint that KHSRP occupies specific genomic sites to regulate the transcription of several hematopoietic genes. Moreover, KHSRP also associates with pre-mRNA intronic regions to modulate alternative splicing during monocytic differentiation. Of note, KHSRP collaboratively controls mago homolog, exon junction complex subunit (MOGOH), and adenosine deaminase RNA-specific B1 (ADARB1) expression at both the transcriptional and posttranscriptional levels. These new data establish a paradigm for analysis of other functional Che-RBPs in different biological systems.

Prof Haojian Zhang is researching the epigenetic transcription regulation of hematopoietic development and blood diseases at Wuhan University, China. Here, Prof Zhang and colleagues compile an interesting review article titled “RNA m^6^A modification: Mapping Methods, Roles and Mechanisms in Acute Myeloid Leukemia.” N6-methyladenosine (m^6^A) is an RNA modification that has important roles in regulating mRNA metabolism, and also implicates in hematologic malignancies, including acute myeloid leukemia (AML). Thanks to the rapid development of high-throughput sequencing technologies, we now have insight into the biological functions of m^6^A modification at the whole transcriptome level. In their review, Zhang et al summarize the latest advances in understanding m^6^A biology, with an emphasis on its machinery and the methods permitting m^6^A mapping. They also discuss the critical roles of RNA m^6^A in AML pathogenesis. Finally, they outline potential AML inhibitors based on targeting m^6^A modifiers that have the potential to become a novel therapeutic approach for affected patients.

Prof Pengxu Qian is conducting research at Zhejiang University, China, on the transcription factors and epigenetic modifications involve in HSC self-renewal and pluripotent differentiation. Deep sequencing technologies have uncovered many small ncRNAs, including snoRNAs, miRNAs, tsRNAs, and circular RNAs, which are highly expressed in HSCs. Prof Qian’s review article titles “Small non-coding RNAs in Regulation of Hematopoietic Stem Cell Homeostasis,” updates us on our emerging understanding of how these small ncRNAs regulate HSC homeostasis. Specifically, the authors summarize the regulatory mechanisms of these small ncRNAs in HSC maintenance. They explain how technical advances are enabling us to dissect the cellular and molecular mechanisms of HSC homeostasis at a new resolution, and hope that the resulting advances in our understanding may lead to the development of new therapeutic strategies against hematopoietic malignancies.

Prof Rui Su is based at the Beckman Research Institute of City of Hope, Monrovia, where she focuses on epitranscriptomics in myeloid malignancies. “Epigenetics” refers to the chemical modifications that govern heritable changes in gene expression, independent of the DNA sequence. By contrast, “epitranscriptomics” describes the covalent decorations in RNA that help govern posttranscriptional gene regulation. Recent advances in high-throughput sequencing and analytical chemistry techniques have identified >100 chemical modifications in RNAs, including N6-methyladenosine (m^6^A), N1-methyladenosine (m^1^A), N6, 2′-O-dimethyladenosine (m^6^A_m_), 5-methylcytidine (m^5^C). Since their identification, many researchers have demonstrate that such mRNA modifications are associated with myeloid malignancies. In their review titled “Epitranscriptomics in Myeloid Malignancie,” Prof Su and colleagues explain the function and regulatory mechanism of the most abundant and best-characterized mRNA modification, m^6^A, combining with other RNA epigenetic marks (A-to-I editing, m^5^C and m^7^C). They then give their expert insight on how we might better elucidate the nature and roles of epitranscriptomic modifications during leukemogenesis.

Prof Dong Wang is working at Southern Medical University, Guangzhou, on the application of computational systems biology techniques to delineate the crucial functions of RNA metabolism in hematopoietic regulation. In their review article, titled “Artificial intelligence and its applications in digital hematopathology,” Prof Dong Wang and colleagues focus on the application of AI to normal and abnormal hematopoietic cell identification. The precise and accurate identification of malignant blood cells is a key step in the diagnosis of blood diseases, such as AML and sickle cell disease. However, the manual morphological examination of blood cells capture on microscope slides is time-consuming and difficult to standardize. AI is an exciting development that is being applied to high-throughput, digital image interpretation; indeed, it is fast becoming the most beneficial technology applied to biomedical imaging. Moreover, the integration of digital images into biological workflows, and the advancement of algorithms and computer vision techniques is expanding the biologist’s horizons beyond the microscope slide. In this review, Prof Wang et al explain key concepts to be aware of in AI, before outlining the recent developments in applying AI to normal or abnormal hematopoietic cell identification. They discuss the great potential of AI over microscopy to improve the resolution, signal and information content of the data obtained, and then speculate on the future directions for the field.
